# Pilot testing an ethanol cornual nerve block as a long-term analgesic for calf disbudding

**DOI:** 10.3168/jdsc.2023-0422

**Published:** 2024-02-01

**Authors:** Alycia M. Drwencke, Sarah J.J. Adcock, Jenifer B. Walker, Cassandra B. Tucker

**Affiliations:** 1 Center for Animal Welfare, Department of Animal Science, University of California, Davis, Davis, CA 95616; 2 Animal Behavior Graduate Group, University of California, Davis, Davis, CA 95616; 3 Department of Animal and Dairy Sciences, University of Wisconsin–Madison, Madison, WI 53706; 4 Kinder Ground, Byrdstown, TN 38549

## Abstract

Disbudding prevents horn growth in calves through thermal or chemical cauterization and causes damage that is painful for weeks following the procedure. Current pain management strategies are only effective from 1 to 2 h (local anesthetic) to 1 to 3 d (nonsteroidal anti-inflammatory drugs). A potential practical solution for addressing longer-term pain may be to administer ethanol as a cornual nerve block. When administered at a high concentration, ethanol damages the functionality of peripheral nerves, promoting localized long-lasting analgesia. It is also thought to be painful, thus ethanol may be combined with lidocaine, as a mixed solution or administered beforehand. We tested the use of an ethanol cornual nerve block for anesthesia around the horn bud in 2 pilot studies. We used different concentrations and amounts of ethanol (100% and 70%) in combination with different ratios of lidocaine in our attempt to identify an effective block. In pilot 1, 14 nondisbudded calves were administered 2 to 4 mL of 100% ethanol below the bony ridge on each side of the head to block the cornual nerve at 3 to 10 d of age (n = 28 horn buds) and observed for 5 wk. The duration of loss of sensation was evaluated using pinprick tests 10 min, 1, 3, and 7 d after the block, and then weekly thereafter until 35 d or full sensation had returned. Pinprick tests consisted of lightly pressing a needle in 10 evenly spaced locations around the base of the horn bud (0 responses = no sensation, 1–5 responses = partial sensation, 6–10 responses = full sensation). Pilot 2 looked at the 24 h after the injection and consisted of 9 nondisbudded calves (5 mL of 2:1, 70% ethanol and 2% lidocaine per horn bud) and 6 disbudded individuals (5 mL of 100% ethanol, 70% ethanol, or 2% lidocaine per horn bud). All treatments were administered at the calf level. Anesthesia was checked 10 min after the injection and 4 or 16 h later. In pilot 1, on the day of the ethanol injection (0 d), there was no sensation in 85% of horn buds. Sensation began to return as early as 1 d after blocking, with only 50%, 21%, and 3% of horn buds having no sensation at 1, 7, and 35 d, respectively. Partial sensation was present in 25%, 17%, and 10% of horn buds at these time points. In pilot 2, 27.8% of horn buds in the nondisbudded group had no sensation, whereas 33.3% had partial sensation 10 min after the injection. In the disbudded calves 10 min after the injection, 100% of horn buds from the 100% ethanol group had partial sensation and 100% of horn buds administered 70% ethanol had full sensation. Four or sixteen hours later, 100% of horn buds had full sensation. Together, in these pilot studies, ethanol provided inconsistent anesthesia when used for a cornual nerve block.

## Highlights

• Disbudding is painful for multiple weeks after the procedure.
• An ethanol cornual nerve block provided inconsistent anesthesia for disbudding in calves.
• Alternative long-term pain mitigation and polled genetics should be explored.

## Graphical Abstract

**Figure:**
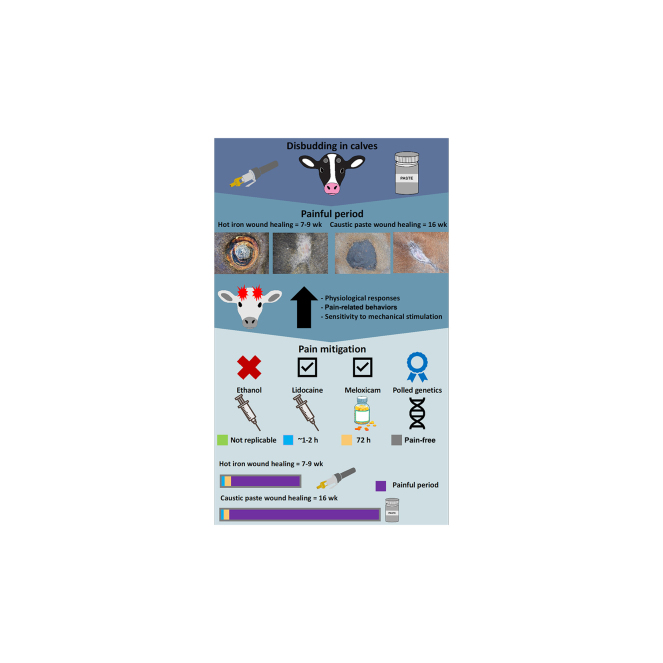
**Summary:** Two methods of disbudding (hot iron and caustic paste) are commonly used. Regardless of the method used, disbudding wounds persist for many weeks, and calves experience acute and long-term pain. Currently available pain mitigation options such as local anesthetics (e.g., lidocaine) and nonsteroidal anti-inflammatory drugs (e.g., meloxicam) are only effective for a few hours to 72 hours. Polled genetics can prevent the pain experienced from disbudding, but is not currently common practice. We tested the use of ethanol as a cornual nerve block to provide longer-term pain relief. As tested in our pilot studies, our results do not indicate that ethanol can provide consistent, replicable pain mitigation.

Disbudding is a common husbandry procedure on dairy farms that prevents horn development with cauterization of free-floating immature horn tissue from a hot iron (heat) or caustic paste (chemical) (USDA, 2018). This procedure is painful in the acute period (Stafford and Mellor, 2011) and throughout the healing process (Adcock and Tucker, 2018; Casoni et al., 2019; Reedman et al., 2022a). Wounds take 7 to 9 wk to re-epithelialize, on average, following hot-iron disbudding (Adcock and Tucker, 2018; Adcock et al., 2019) and 16 wk following caustic paste application (Drwencke et al., 2023a). During this time, calves experience increased sensitivity to touch until wounds re-epithelialize (Adcock and Tucker, 2018; Drwencke et al., 2023a), pain-related behaviors at least 11 d later (Adcock et al., 2020), decreased rumination for 11 d (Adcock et al., 2023), conditioned place preference for pain mitigation 3 wk after the procedure (Adcock and Tucker, 2020), place aversion after 48 h (Ede et al., 2020), and more shelter use for at least 3 d (Gingerich et al., 2020). Together, these studies highlight the need for longer lasting pain relief following disbudding. However, current best practice to mitigate pain following disbudding is a combination of a nonsteroidal anti-inflammatory drug and local block (Reedman et al., 2022b), which provides relief for up to 72 h at best (Mosher et al., 2012).

In humans, ethanol blocks are a well-established therapy for intractable, chronic pain (Jackson and Gaeta, 2008). When administered at a high concentration (typically >98%), ethanol damages myelin, the fatty sheath surrounding nerves, disrupting electrical impulse conduction involved in pain perception, promoting long-lasting analgesia in that region (Guerri and Pascual, 2016). A single injection is effective at alleviating pain for at least 1 yr in peripheral nerve disorders such as trigeminal neuralgia (Han et al., 2017) and Morton's neuroma (Pasquali et al., 2015). Administering lower concentrations of ethanol has been studied in pigs for pancreatic tissue ablation and was found to reduce bordering tissue damage (Matthes et al., 2007). Ethanol is reported to cause pain during the injection, but this effect can be mitigated by administering local anesthesia beforehand (Wiersema and Wiersema, 1996). Administering a lower concentration of ethanol simultaneously with a local anesthetic could reduce the number of injections while potentially reducing both acute and long-term pain.

Previous work in livestock shows ethanol blocks alleviate chronic rectal tenesmus in cattle for up to 5 wk with no adverse side effects (Noordsy, 1982). In horses, ethanol injections are considered a safe and economical treatment for clinical cases of osteoarthritis (70% or 100% ethanol: Lamas et al., 2012; Caston et al., 2013). Ethanol has also been used to achieve renal sympathetic denervation in sheep and pigs (Fischell et al., 2013; 99.6% ethanol: Firouznia et al., 2015). Administering an ethanol cornual nerve block was first demonstrated to numb the horn bud to mechanical stimulation for 3 d and to pinpricks for up to 178 d after disbudding (100% ethanol: Tapper, 2011). Similarly, local infiltration of ethanol was recently examined for dehorning in 20-wk-old cattle (100% ethanol and lidocaine cornual nerve block: Martin et al., 2022).

Administering ethanol as a cornual nerve block before disbudding has some advantages over other analgesics: (1) it is administered using the same technique as a lidocaine block, (2) there are no residue concerns (personal communication, Food Animal Residue Avoidance Databank, Davis, CA, Case Number VA-06022019-161504; R. Van Vleck Pereira), and (3) a single dose could relieve both acute and long-term pain. Adverse symptoms described in humans include inflammation at the injection site and excessive or abnormal sensation such as tingling, pricking, numbness, or burning. In cattle, untargeted structures around the cornual nerve could be harmed, causing permanent drooping of the eyelid. Our objective was to conduct a preliminary investigation into the use of an ethanol cornual nerve block for potential acute and long-term pain relief in young dairy calves. We aimed to better understand the duration of anesthesia from ethanol from administration through return of sensation for calves and gain experience with this tool in multiple contexts such as different facilities and age groups, across concentrations (70%, 100%), and when combined with lidocaine.

Data were collected in a series of 2 pilot studies. Pilot 1 took place at the University of California–Davis Dairy Teaching and Research Facility between June 2019 and April 2020. The University of California–Davis Institutional Animal Care and Use Committee approved procedures during this phase (protocol #21170). Pilot 2 was run on a commercial farm in central California in July 2021 under the supervision of a licensed veterinarian. Treatments and sample size for each pilot study are outlined in Table 1. Total sample size was opportunistic and not determined by a power analysis. Researchers were not blind to treatment. All data and supplemental figures are available in the Dryad repository (Drwencke et al., 2023b,c).

**Table 1:** Treatment description of the amount and type of ethanol used, sample size, calf age, and whether calves were disbudded before the injection or not

Group	Treatment	Sample	Age when injected (d)	Disbudded at the time of injection?
Pilot 1				
1	2 mL of 100% ethanol, additional 1 mL if not numb after 10 min	3 Jerseys	3–4	No
2	3 mL of 100% ethanol, additional 1 mL if not numb after 10 min	2 Jerseys, 1 Holstein	3–4	No
3	3 mL of 100% ethanol, additional 1 mL if not numb after 10 min	1 Jersey, 3 Holsteins	9–10	No
4	3 mL of buffered lidocaine, followed 10 min later with 4 mL of 100% ethanol	4 Holsteins	9–10	No
Pilot 2				
1	5 mL of mixed 2% lidocaine and 70% ethanol (2:1 ratio)	9 Holsteins	1–4	No
2	5 mL of 2% lidocaine only	2 Holsteins	9–8	Yes
3	5 mL of 100% ethanol	2 Holsteins	12–13	Yes
4	5 mL of 70% ethanol	2 Holsteins	10–11	Yes

In pilot 1, 14 nondisbudded heifer calves (n = 6 Jersey and 8 Holstein) were housed individually in wire panel pens (2.5 × 1.2 × 0.9 m; length × width × height) bedded with rice hulls (~15–20 cm depth). Ad libitum access to water and grain (Starter Calf Feed 901033, Associated Feed and Supply Co.) was provided. Milk was fed twice daily as described by Drwencke et al. (2023a) and calves were stepped up to 6 L/d by 32 d of age.

In pilot 1, 100% ethanol was injected subcutaneously as a cornual nerve block (Table 1). Groups 1 to 3 were administered 2 or 3 mL per side of 100% ethanol only. Group 4 was injected with 3 mL of buffered lidocaine, followed 10 min later by 4 mL of 100% ethanol in an effort to mitigate potential injection pain. During all injections, calves were placed in a head restraint in the home pen (see Jimenez et al., 2019). A 12-mL syringe with a 20-gauge × 25-mm needle was injected at a 45° angle toward the horn bud in the divot below the bony ridge that runs between the eye and temple. The needle was inserted to the hub, aspirated to confirm it was not in a blood vessel, and approximately half of the ethanol was injected with a side-to-side motion of the syringe. The needle was withdrawn approximately halfway, and the remainder of the ethanol was injected. Calves were checked for anesthesia 10 min after the injection and an additional 1 mL was provided if a behavioral response occurred (4 horn buds from 4 calves). Local swelling was observed after the first calf received ethanol injections. This calf was given 1 mg/kg meloxicam and thereafter the remaining 13 calves in pilot 1 received ~1 mg/kg meloxicam approximately 1 h before ethanol injections.

To evaluate anesthesia following injections, a clean needle was used to gently prick around the base of the horn buds while we monitored for a behavioral reaction (ear flick or pulling the head away). Pinpricks occurred in 10 evenly spaced locations (0 responses = no sensation, 1–5 responses = partial sensation, 6–10 responses = full sensation/bud). If a response occurred in any location, that spot was re-tested to confirm sensation. In pilot 1, calves had a pin prick test performed 10 min and 1, 3, 7, 14, 21, 28, and 35 d after the injection. At these same time points, calves were visually monitored for adverse side effects of the ethanol injections including swelling at the injection site and drooping eyelids. All pinpricks and assessments were conducted by a single person. Once a horn bud had 10 responses to a pinprick, it was not tested at future time points and assumed to have full sensation.

The R (version 4.0.3 on macOS Big Sur 10.16) software was used to create descriptive summaries and Figure 1 (R Core Team, 2022). One calf was missing a d 3 pinprick; calves that received lidocaine were excluded from 0 d results to attribute loss of sensation solely to ethanol. Ethanol initially resulted in no sensation for 85% of horn buds in pilot 1. Sensation began to return as early as 1 d after the injection. No sensation was present in only 3.5% of horn buds 35 d later. At 35 d after injection, 10.7% of horn buds had partial sensation (Figure 1). No drooping eyelids were observed at any point. Swelling at the site of the injection (presence or absence; Supplemental Figures S1 and S2, Drwencke et al., 2023c) was observed and potential tissue hardening (“crunchy” texture; presence or absence) was detected multiple weeks after ethanol administration when a new lidocaine block was given before disbudding these calves.

**Figure 1:**
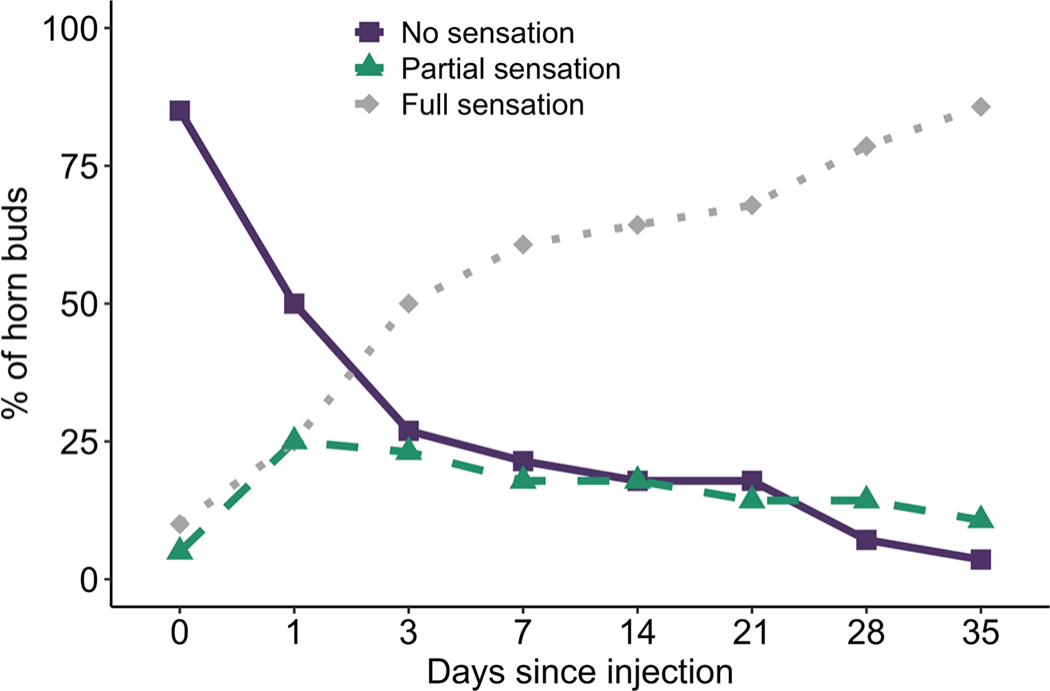
Percentage of horn buds (n = 28) with no, partial, or full sensation during pilot 1 following the injection of 100% ethanol as a cornual nerve block. Anesthesia was evaluated with gentle needle pinpricks around 10 locations at the base of the horn bud 10 min and 1, 3, 7, 14, 21, 28, and 35 d after the injection. Calves injected with lidocaine were excluded from 0 d values. Horn buds were considered to have no sensation when there were no behavioral responses and partial sensation with 1 to 5 reactions (out of 10).

In pilot 2, 15 Holstein heifer calves were housed in individual plastic-sided pens (0.75 × 1.5 × 1 m; length × width × height) located within a curtain-sided barn. Straw bedding ~0.5 m in depth was provided for approximately 1 wk; then plastic slatted flooring remained. Access to water and starter were available ad libitum. Calves were fed 3.8 L of colostrum at birth followed by 2 L of transition milk 2 times/d through 2 d of age. Starting at 3 d, calves were fed 2.8 L of pasteurized whole milk 2 times/d via a bottle. Ad libitum access to water and grain (Ultimate Calf Starter 205111, Associated Feed and Supply Co.) was provided from birth.

In pilot 2, 4 approaches were tested (Table 1), including a lower concentration of ethanol in an effort to mitigate the swelling and tissue hardening observed in pilot 1. Treatments were administered consistently within a calf and allocated first based on available nondisbudded calves (group 1), then with similarly aged calves (groups 2–4). All calves in pilot 2 were restrained so the shoulder blades were between a human's legs, and the head was braced but the calf could drink from the provided milk bottle. Group 1 consisted of nondisbudded calves (n = 9) that received a 2:1 ratio of 2% lidocaine and 70% ethanol mixed in a 25-mL automatic injection syringe (Agri-Pro Enterprises). Lidocaine was drawn into the syringe first, followed by ethanol. Five milliliters per horn bud was administered (1.7 mL of ethanol, 3.4 mL of lidocaine) using a 20-gauge × 12.5-mm needle. The mixture was injected subcutaneously with the needle inserted to the hub and angled 45° toward the horn bud in the divot below the bony ridge. The other 3 groups were calves (n = 2/group) that had previously been disbudded using Dr. Naylor's caustic paste (H. W. Naylor Company Inc.) by the farm employees at 3 to 5 d of age. Disbudded calves were used to test unmixed injections within this pilot opportunistically since there were no additional nondisbudded calves available. The disbudded groups were injected using a 20-gauge × 12.5-mm needle attached to a 12-mL syringe. For each of these groups, 2 calves were injected with 5 mL of 100% ethanol, 70% ethanol, or 2% lidocaine per horn bud. Pilot 2 calves were provided ~1 mg/kg meloxicam before injections.

A pinprick test was used to assess anesthesia as described in pilot 1 for all groups. Restraint was performed in a similar fashion to the injections, with no bottle, and the head was not fully restricted. All calves had a pin prick test performed 10 min after their injection, group 1 had another 4 h later. Pin pricks were conducted the same day for group 1 (mixed lidocaine and ethanol). When 100% of calves had full sensation at 4 h, groups 2–4 were injected with lidocaine, or ethanol (70% or 100%) and checked for anesthesia. Groups 3 and 4 were tested a second time 16 h later. All pinpricks were performed by a single person trained by the researcher from pilot 1. For ethical reasons, during 6 tests (10% of total tests) fewer than 10 pinpricks were used to assess anesthesia when calves had full sensation and showed escape attempts. Injection site swelling was observed at 15 sites across 9 pilot 2 calves, but no drooping eyelids occurred. After data collection, nondisbudded calves were provided lidocaine and meloxicam before hot-iron (pilot 1) or paste (pilot 2) disbudding.

Results for pilot 2 are shown in Table 2; all animals were included. Desensitization was poor across groups with the exception of short-term loss of sensation for the lidocaine only group. Notably, the lidocaine is likely contributing to the sensation level in the mixed injection group at the 10-min observation. It is unclear if mixing the lidocaine and ethanol or total volume injected (5 mL total; 1.7 mL of ethanol, 3.4 mL of lidocaine) led to high levels of sensation.

**Table 2:** Percentage of horn buds with no or partial sensation during pilot 2^1^

Group	Injection type	Horn buds (no.)	No sensation 10 min (%)	Partial sensation 10 min (%)	No sensation 4+ h (%)	Partial sensation 4+ h (%)
1	5 mL of mixed 2% lidocaine and 70% ethanol (2:1 ratio)	18	27.8	33.3	0	0
2	5 mL of 2% lidocaine only^2^	4	100	0	NA^3^	NA
3	5 mL of 100% ethanol	4	0	100	0	0
4	5 mL of 70% ethanol	4	0	0	0	0

1 Anesthesia was evaluated with gentle needle pinpricks around 10 locations at the base of the horn bud 10 min and 4 or 16 h after the injection. Horn buds were considered to have no sensation when there were no behavioral responses and partial sensation if there were 1 to 5 reactions (out of 10).

2 Sensation was tested only at the 10-min time point because lidocaine only lasts for 2 to 3 h.

3 NA = data that were not collected, as lidocaine is known to be ineffective 4 h after administration.

In our 2 pilot studies, ethanol provided inconsistent anesthesia when used for a cornual nerve block. These results contrast with Tapper (2011) who found that ethanol provided consistent anesthesia in dairy calves for up to 178 d following the injection. However, Martin et al. (2022) found local infiltration of ethanol did not extend pain mitigation beyond that of lidocaine and meloxicam. While it is unclear what causes the inconsistency, it is possible ethanol concentration, age, nerve regeneration, tissue quantity, or injection location could play a role. Taken together, our results along with the work of others (Tapper, 2011; Martin et al., 2022) suggests that using ethanol as a cornual nerve block creates variable results and does not provide long-term pain relief for calves.

In pilot 2, we gave 4 calves pure ethanol injections while 9 calves received a mix of 70% ethanol and lidocaine 2% and achieved only 33% loss of sensation. In contrast, we had 85% loss of sensation in pilot 1, where all calves received 100% ethanol. Concentrations of ethanol as low as 20% to 40% have been reported to create local anesthetic effects in humans (Ritchie, 1996; Zafonte and Munin, 2001). Notably, human studies used lower concentrations in more tissue-dense regions such as the spine and thigh (May et al., 1912; Zafonte and Munin, 2001). The same is true when lower concentrations of ethanol were injected into the pancreas of pigs (Matthes et al., 2007).

To prevent sensation, the cornual nerve must be sufficiently damaged by ethanol. The low quantity of tissue surrounding the cornual nerve may have prevented the ethanol from being held in place to create long-lasting damage. Fierheller et al. (2012) suggested their ineffective use of local anesthetic with a percutaneous jet delivery administration could have been due to a lack of underlying soft tissue, or inadequate drug concentrations around the cornual nerve. In other animals where ethanol was effective, more soft tissue was present near the injection than what surrounds the cornual nerve. For example, ethanol was injected into the spine in cattle (Noordsy, 1982; Valverde and Sinclair, 2015), intra-articularly in horses (Lamas et al., 2012; Caston et al., 2013), and into the renal arteries in sheep and pigs (Fischell et al., 2013; Firouznia et al., 2015). Indeed, onset of the cornual nerve block relies on the placement and proximity of the anesthetic to the nerve, with delay or failure associated with deeper administration in the muscle rather than surrounding the branches (Skarda, 1996; Anderson and Edmondson, 2013; Bates et al., 2019).

Bates et al. (2019) attribute some possible differences in anesthesia to administrator skill with the technique, but even with ethanol injections that achieved no sensation at the 10-min check, the length of anesthesia was inconsistent. Studies that have used a lidocaine cornual nerve block have reported a wide range of success (reviewed by Sheedy et al., 2024). These vary from 55% (Bates et al., 2019) to 75% to 91% with newly trained individuals (Winder et al., 2018) and 87.5% (Fierheller et al., 2012) achieving full anesthesia. The individuals performing ethanol injections (SJJA and AMD) have administered ~1,800 lidocaine cornual nerve blocks with ~12% needing more than one dose to achieve full anesthesia. Although we cannot rule out a potential influence of administrator technique, the inconsistent duration of anesthesia, insufficient numbness, and potential negative side effects support the need for other approaches to pain relief.

Our pilot studies include several confounding factors, including age, breed, and disbudding status; we also used several ethanol concentrations, including a range of how this was combined with lidocaine. Through this mixed approach, which was pilot in nature, we found that we could not consistently create an effective block with ethanol. This step would be needed to pursue more formal testing of this approach. However, we identified swelling and possible tissue damage, concerning negative side effects. For us, the inconsistent results and negative side effects support exploring other alternative forms of anesthesia and polled genetics to mitigate or prevent pain throughout the disbudding healing process.

## References

[bib1] Adcock S.J.J., Cruz D.M., Tucker C.B. (2020). Behavioral changes in calves 11 days after cautery disbudding: Effect of local anesthesia. J. Dairy Sci..

[bib2] Adcock S.J.J., Downey B.C., Owens C., Tucker C.B. (2023). Behavioral changes in the first 3 weeks after disbudding in dairy calves. J. Dairy Sci..

[bib3] Adcock S.J.J., Tucker C.B. (2018). The effect of disbudding age on healing and pain sensitivity in dairy calves. J. Dairy Sci..

[bib4] Adcock S.J.J., Tucker C.B. (2020). Conditioned place preference reveals ongoing pain in calves 3 weeks after disbudding. Sci. Rep..

[bib5] Adcock S.J.J., Vieira S.K., Alvarez L., Tucker C.B. (2019). Iron and laterality effects on healing of cautery disbudding wounds in dairy calves. J. Dairy Sci..

[bib6] Anderson D.E., Edmondson M.A. (2013). Prevention and management of surgical pain in cattle. Vet. Clin. North Am. Food Anim. Pract..

[bib7] Bates A.J., Sutherland M.A., Chapple F., Dowling S.K., Johnson A.P., Saldias B., Singh J. (2019). A new method of administering local anesthesia for calf disbudding: Findings from a comparative on-farm study in New Zealand. J. Dairy Sci..

[bib8] Casoni D., Mirra A., Suter M.R., Gutzwiller A., Spadavecchia C. (2019). Can disbudding of calves (one versus four weeks of age) induce chronic pain?. Physiol. Behav..

[bib9] Caston S., McClure S., Beug J., Kersh K., Reinertson E., Wang C. (2013). Retrospective evaluation of facilitated pastern ankylosis using intra-articular ethanol injections: 34 cases (2006–2012). Equine Vet. J..

[bib10] Drwencke A.M., Adcock S.J.J., Tucker C.B. (2023). Wound healing and pain sensitivity following caustic paste disbudding in dairy calves. J. Dairy Sci..

[bib11] Drwencke A.M., Adcock S.J.J., Walker J.B., Tucker C.B. (2023).

[bib12] Drwencke A.M., Adcock S.J.J., Walker J.B., Tucker C.B. (2023). Data from: Pilot testing an ethanol cornual nerve block as a long-term analgesic for calf disbudding. Zenodo.

[bib13] Ede T., von Keyserlingk M.A.G., Weary D.M. (2020). Conditioned place aversion of caustic paste and hot-iron disbudding in dairy calves. J. Dairy Sci..

[bib14] Fierheller E.E., Caulkett N.A., Haley D.B., Florence D., Doepel L. (2012). Onset, duration and efficacy of four methods of local anesthesia of the horn bud in calves. Vet. Anaesth. Analg..

[bib15] Firouznia K., Hosseininasab S.J., Amanpour S., Haj-Mirzaian A., Miri R., Muhammadnejad A., Muhammadnejad S., Jalali A.H., Ahmadi F., Rokni-Yazdi H. (2015). Renal sympathetic denervation by CT-scan-guided periarterial ethanol injection in sheep. Cardiovasc. Intervent. Radiol..

[bib16] Fischell T.A., Vega F., Raju N., Johnson E.T., Kent D.J., Ragland R.R., Fischell D.R., Almany S.L., Ghazarossian V.E. (2013). Ethanol-mediated perivascular renal sympathetic denervation: Preclinical validation of safety and efficacy in a porcine model. EuroIntervention.

[bib17] Gingerich K.N., Choulet V., Miller-Cushon E.K. (2020). Disbudding affects use of a shelter provided to group-housed dairy calves. J. Dairy Sci..

[bib18] Guerri C., Pascual M., Preedy V. (2016). Neuropathology of Drug Addictions and Substance Misuse.

[bib19] Han K.R., Chae Y.J., Lee J.D., Kim C. (2017). Trigeminal nerve block with alcohol for medically intractable classic trigeminal neuralgia: Long-term clinical effectiveness on pain. Int. J. Med. Sci..

[bib20] Jackson T.P., Gaeta R. (2008). Neurolytic blocks revisited. Curr. Pain Headache Rep..

[bib21] Jimenez R.E., Adcock S.J.J., Tucker C.B. (2019). Acute pain responses in dairy calves undergoing cornual nerve blocks with or without topical anesthetic. J. Dairy Sci..

[bib22] Lamas L.P., Edmonds J., Hodge W., Zamora-Vera L., Burford J., Coomer R., Munroe G. (2012). Use of ethanol in the treatment of distal tarsal joint osteoarthritis: 24 cases. Equine Vet. J..

[bib23] Martin M.S., Kleinhenz M.D., Viscardi A.V., Montgomery S.R., Cull C.A., Seagren J.E., Lechtenberg K.F., Coetzee J.F. (2022). Comparison of lidocaine alone or in combination with a local nerve block of ethanol, bupivacaine liposome suspension, or oral meloxicam to extend analgesia after scoop dehorning in Holstein calves. JDS Commun..

[bib24] Matthes K., Mino-Kenudson M., Sahani D.V., Holalkere N., Brugge W.R. (2007). Concentration-dependent ablation of pancreatic tissue by EUS-guided ethanol injection. Gastrointest. Endosc..

[bib25] May O., Cantab M.D., Lond M.R.C.P. (1912). The functional and histological effects of intraneural and intraganglionic injections of alcohol. BMJ.

[bib26] Mosher R.A., Coetzee J.F., Cull C.A., Gehring R., Kukanich B. (2012). Pharmacokinetics of oral meloxicam in ruminant and preruminant calves. J. Vet. Pharmacol. Ther..

[bib27] Noordsy J.L. (1982). Sacral paravertebral alcohol nerve block as an aid in controlling chronic rectal tenesmus in cattle. Vet. Med. Small Anim. Clin..

[bib28] Pasquali C., Vulcano E., Novario R., Varotto D., Montoli C., Volpe A. (2015). Ultrasound-guided alcohol injection for Morton’s neuroma. Foot Ankle Int..

[bib29] R Core Team (2022). R: A language and environment for statistical computing. https://www.r-project.org/.

[bib30] Reedman C.N., Duffield T.F., DeVries T.J., Lissemore K.D., Adcock S.J., Tucker C.B., Parsons S.D., Winder C.B. (2022). Effect of plane of nutrition and analgesic drug treatment on wound healing and pain following cautery disbudding in preweaning dairy calves. J. Dairy Sci..

[bib31] Reedman C.N., Duffield T.F., DeVries T.J., Lissemore K.D., Winder C.B. (2022). Graduate Student Literature Review: Role of pain mitigation on the welfare of dairy calves undergoing disbudding. J. Dairy Sci..

[bib32] Ritchie J., Harpman J., Limbirol G. (1996). Goodman and Gilman’s: The Pharmacological Basis of Therapeutics.

[bib33] Sheedy D.B., Aly S.S., Tucker C.B., Lehenbauer T.W. (2024). Mini-review: The history and future of the cornual nerve block for calf disbudding. JDS Commun..

[bib34] Skarda R.T. (1996). Local and regional anesthesia in ruminants and swine. Vet. Clin. North Am. Food Anim. Pract..

[bib35] Stafford K.J., Mellor D.J. (2011). Addressing the pain associated with disbudding and dehorning in cattle. Appl. Anim. Behav. Sci..

[bib36] Tapper K.R. (2011).

[bib37] USDA. 2018. Health and Management Practices on U.S. Dairy Operations, 2014. Fort Collins, CO.

[bib38] Valverde A., Sinclair M., Grimm K.A.G., Lamont L.A., Tranquilli W.J., Greene S.A., Robertson S.A. (2015). Veterinary Anesthesia and Analgesia: The Fifth Edition of Lumb and Jones.

[bib39] Wiersema M.J., Wiersema L.M. (1996). Endosonography-guided celiac plexus neurolysis. Gastrointest. Endosc..

[bib40] Winder C.B., LeBlanc S.J., Haley D.B., Lissemore K.D., Godkin M.A., Duffield T.F. (2018). Comparison of online, hands-on, and a combined approach for teaching cautery disbudding technique to dairy producers. J. Dairy Sci..

[bib41] Zafonte R.D., Munin M.C. (2001). Phenol and alcohol blocks for the treatment of spasticity. Phys. Med. Rehabil. Clin. N. Am..

